# Comparison of Three Untargeted Data Processing Workflows for Evaluating LC-HRMS Metabolomics Data

**DOI:** 10.3390/metabo10090378

**Published:** 2020-09-21

**Authors:** Selina Hemmer, Sascha K. Manier, Svenja Fischmann, Folker Westphal, Lea Wagmann, Markus R. Meyer

**Affiliations:** 1 Department of Experimental and Clinical Toxicology, Institute of Experimental and Clinical Pharmacology and Toxicology, Center for Molecular Signaling (PZMS), Saarland University, 66421 Homburg, Germany; selina.hemmer@uks.eu (S.H.); sascha.manier@uks.eu (S.K.M.); lea.wagmann@uks.eu (L.W.); 2 State Bureau of Criminal Investigation Schleswig-Holstein, 24116 Kiel, Germany; Svenja.Dr.Fischmann@polizei.landsh.de (S.F.); Folker.Dr.Westphal@polizei.landsh.de (F.W.)

**Keywords:** untargeted metabolomics, LC-HRMS, data processing, feature detection, A-CHMINACA

## Abstract

The evaluation of liquid chromatography high-resolution mass spectrometry (LC-HRMS) raw data is a crucial step in untargeted metabolomics studies to minimize false positive findings. A variety of commercial or open source software solutions are available for such data processing. This study aims to compare three different data processing workflows (Compound Discoverer 3.1, XCMS Online combined with MetaboAnalyst 4.0, and a manually programmed tool using R) to investigate LC-HRMS data of an untargeted metabolomics study. Simple but highly standardized datasets for evaluation were prepared by incubating pHLM (pooled human liver microsomes) with the synthetic cannabinoid A-CHMINACA. LC-HRMS analysis was performed using normal- and reversed-phase chromatography followed by full scan MS in positive and negative mode. MS/MS spectra of significant features were subsequently recorded in a separate run. The outcome of each workflow was evaluated by its number of significant features, peak shape quality, and the results of the multivariate statistics. Compound Discoverer as an all-in-one solution is characterized by its ease of use and seems, therefore, suitable for simple and small metabolomic studies. The two open source solutions allowed extensive customization but particularly, in the case of R, made advanced programming skills necessary. Nevertheless, both provided high flexibility and may be suitable for more complex studies and questions.

## 1. Introduction 

Metabolomics is defined as the analysis of the whole metabolome of a biological system and therefore, aims to detect as many metabolites as possible in a biological sample [1,2]. However, the metabolic profile is not limited to endogenous metabolites but also metabolites of exogenous sources like drugs, diet, and gut microbiota may be added in. Furthermore, metabolomic studies can be divided into two major strategies, untargeted and targeted approaches. Targeted metabolomics usually aims to detect and quantify specific metabolites of known structures. The untargeted or global approach usually aims to identify as many metabolites as possible without having any previous knowledge about them [1,3].

Due to its high selectivity and sensitivity, liquid chromatography coupled to high-resolution mass spectrometry (LC-HRMS) is currently the most commonly applied analytical technique in metabolomics [4,5,6]. To correctly interpret differences in specific metabolites and to gain a proper biological interpretation, a reliable and suitable entire approach is necessary [4,5]. The part of data processing includes a series of steps such as peak detection, peak alignment, baseline correction, and annotation [7,8,9]. Data processing of LC-HRMS raw data is a key step in untargeted metabolomic studies, which establishes a sound basis to accurately identify significant changes. It involves reducing the complexity of raw data by extracting features, and usually transforming them in order to subsequently perform adequate statistical tests [9,10].

A variety of software solutions are available for untargeted data processing, such as the open source software XCMS, MZmine, OpenMS [11], MetAlign, MetaboAnalyst [12] and the commercial software MarkerView, Compound Discoverer (CD), MetaboScape etc. In the case of open source software, modules are often based on the programming language R [7]. 

Since the underlying algorithms differ, it is very likely that the outcome of a metabolomic study might vary upon the tools used. Li et al. compared the performance of five software solutions (MS-Dial, MZmine, XCMS, MarkerView, and CD) on a benchmark dataset from standard mixtures. All five software solutions revealed similar performance in detecting true features. Nevertheless, to select true discriminating markers, they recommended the combination of MZmine 2 and XCMS [13]. Fernández-Ochoa et al. determined that Agilent Profinder showed good quality of the graphs and was characterized by its ease of use, whereas the R pipeline seemed to be better suited for studies with a large number of samples [7].

Since further studies are missing and the selection of an appropriate tool is essential for the quality and outcome of the statistical evaluation, the present study aimed to compare three different data processing workflows to investigate LC-HRMS data of an untargeted metabolomics study, namely the commercially available software CD 3.1, the open source online tool XCMS Online in combination with MetaboAnalyst 4.0 (XCMS/MetaboAnalyst), and a manually programmed tool using the language R based on different R packages [14]. XCMS, MetaboAnalyst, and the R script were chosen as they were identified as suitable and were successfully used in previous studies [15,16,17]. Simple but highly standardized datasets for evaluation should be used by incubating pooled human liver microsomes (pHLM) with the synthetic cannabinoid A-CHMINACA (1(-cyclohexylmethyl)-*N*-tricyclo[3.3.1.1^3,7^]dec-1-yl-1H-indazole-3-carboxamide). The outcome of each workflow should be evaluated by its number of significant features, the quality of the peaks, and the results of multivariate statistics. Additionally, the metabolite profile of A-CHMINACA in pHLM should be elucidated. 

## 2. Results and Discussion 

### 2.1. Study Design

Due to the ease of use and low variability of individual pHLM incubations and the fact that it is a very well characterized in vitro model for drug metabolism studies, incubations of pHLM with the synthetic cannabinoid A-CHMINACA were prepared to generate simple datasets [18]. The incubation mixtures were then analyzed using LC-HRMS/MS and finally, three different software tools for untargeted data processing were applied to identify significant features. Software evaluation in this study included the commercial software CD 3.1, which was developed for the used type of MS instrument; open source software workflows including a combination of XCMS Online and MetaboAnalyst 4.0; and a manually programmed tool using R. While XCMS-based software tools might be one of the best solutions for LC-HRMS/MS untargeted metabolomics, XCMS was used as a preprocessing tool in the case of the two open source workflows [15,19,20,21,22]. After data processing, significant features were identified and the metabolic fate of A-CHMINACA in pHLM was elucidated. The three untargeted data processing workflows were extensively evaluated with regards to their number of significant features, the peak quality of the significant feature, their false positive rate, and the results of the multivariate statistics. 

### 2.2. Untargeted Metabolomics

#### 2.2.1. Parameter Optimization for the Three Different Workflows of Untargeted Metabolomics 

In untargeted data processing, the optimization of various parameters is important to allow for the detection of chromatographic peaks, construct extracted ion chromatograms (EICs), annotate features, and for chromatogram alignment [19]. Since the two open source software tools XCMS/MetaboAnalyst and R are not already optimized, peak picking and alignment parameters were optimized using a previously optimized workflow [15]. The optimized XCMS parameters are summarized in Table S1. Using the R workflow, all eight parameters could be transferred in exactly the same way. For XCMS Online, prefilter 1 was limited up to 10. If this parameter was greater than 10, 10 was used for XCMS Online. Additionally, the parameter bandwidth could only be specified in positive integer numbers in XCMS Online, so if this parameter was less than 1, 1 was used. Since the commercial software CD was developed specifically in combination with the used MS instrument type, an already existing workflow for untargeted metabolomics, namely “Untargeted Metabolomics with statistics detect unknowns with ID using Online Database and mzLogic”, was chosen without changing any parameters.

#### 2.2.2. Comparison of Significant Features of the Three Different Workflows 

**Figure 1:**
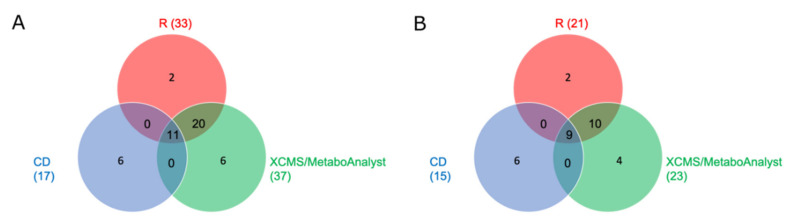
Comparison of the significant features of the three data processing workflows R (red), XCMS Online combined with MetaboAnalyst (green), and Compound Discoverer (CD, blue) displayed as Venn Diagram; (**A** = with isotopes and adducts; **B** = without isotopes and adducts).

Univariate statistics was done using one-way analysis of variance (ANOVA) for all three workflows (Figure S1). False positive results were prevented using Bonferroni correction as a multiple testing correction technique [23]. Since the settings of XCMS Online did not allow a change from the Kruskal–Wallis test to ANOVA in multi-group comparisons for no evident reason, XCMS Online was only used for peak picking and alignment. The resulting table was then reduced to the peak areas and retention times between 1 and 10 min. The entire statistical evaluation was performed using MetaboAnalyst 4.0. Visual inspection of the plotted ANOVA results (Figure S1) of the two open source workflows revealed that they were similar to each other concerning significant features and their corresponding *p*-values. In contrast to this, differential analysis over all three groups was not possible using CD, because the software does not allow one to do a statistical evaluation of more than two groups. Thus, statistical evaluation using a Welch *t*-test in combination with the corresponding fold change had to be performed for blank vs. low, blank vs. high, and low vs. high. A feature was considered significant if it was significant between one of the two groups. In terms of number of significant features, 15 significant features were obtained for CD, 32 for the XCMS/MetaboAnalyst solution, and 28 for R using normal phase chromatography and positive ionization mode. In the case of using a reversed phase chromatography and positive ionization mode, 5 significant features were received for CD, 13 for XCMS/MetaboAnalyst, and 11 for R. None of the analyses indicated significant features using negative ionization mode. The Venn diagram in Figure 1A shows the composition of all significant features obtained after using the three data processing workflows and the two analytical columns. In total, 11 of the significant features were detected after using each of the three workflows, 31 significant features were determined after using both open source workflows, and 17 after using CD. While the manually programmed R tool used the R package CAMERA to identify isotopes and adducts in the dataset, CD annotated neither isotopes nor adducts. In CD, isotopes and adducts were merely labeled in the spectrum of the related compound, but not listed in the compound list and therefore, not annotated as significant features. Taking this into account, the number of significant features identified by the two open source workflows that are neither isotopes nor adducts could be reduced to 9 (Figure 1B). 

In addition to the number of significant features, the three workflows were also evaluated according to the peak shape quality of these features. Since the extracted ion chromatogram (EIC) of some detected significant features appeared to be false positive hits, the significant features were divided into true and false features based on the peak shape quality of their EIC. Therefore, peak quality was divided into two main categories. The first category included non-existent group differences, which means that in the EIC of the respective significant features, there was no clear separation of peak intensity between the four groups Blank, Low, High, and QC. The second category included the non-correct peak integration, which means that in the EIC, the integrated peak could not be separated from the baseline. By comparing the quality of the peaks based on the two categories mentioned above, the overall true features for the three different workflows were 17 for CD, 28 for XCMS Online/MetaboAnalyst, and 24 for R. The significant features detected by CD were all identified as true features, which can be explained by the fact that this workflow does not show isotopes or adducts as significant features. Furthermore, in comparison to the two open source workflows, CD used a fold change of 1 in addition to the *p*-value in order to filter the features in one of the group comparisons. The true features are listed in Table 1 and Table 2.

#### 2.2.3. Comparison of Multivariate Statistics of the Three Different Software Workflows

In addition to univariate statistics, datasets are usually also analyzed using multivariate methods to identify the largest changing features and specific signatures in the data [2]. In this study, principal component analysis (PCA) and hierarchical clustering were used to evaluate differences between the three workflows. 

**Figure 2:**
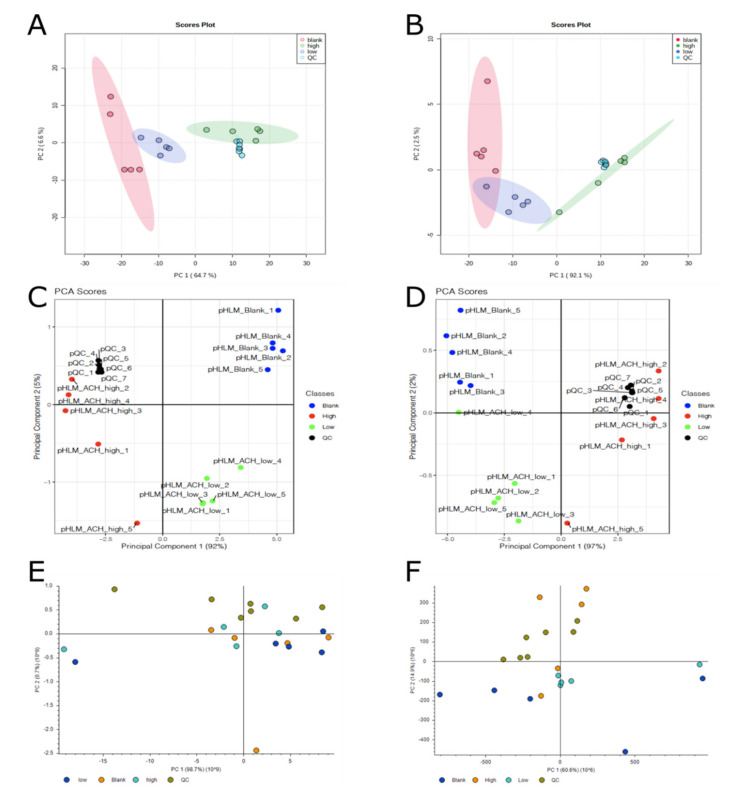
Scores of principal component analysis. (**A** = XCMS Online/MetaboAnalyst, HILIC column, positive mode; **B** = XCMS Online/MetaboAnalyst, PhenylHexyl column, positive mode; **C** = R, HILIC column, positive mode; **D** = R, PhenylHexyl column, positive mode; **E** = Compound Discoverer, HILIC column, positive mode; **F** = Compound Discoverer, PhenylHexyl column, positive mode).

PCA, as a non-supervised method, does not use any group information to find the principal component. It is a data reduction technique, which enables high dimensional datasets to be reduced to a few major principal components (PC) [24]. The scores of these components, which are the weighted sum of the contribution of each metabolite to a principal component, are plotted. It can be seen that each incubation group is distinct from another one. In addition to the score plot, the loading plot provides information on which metabolites are contributing the most to the separations between groups [24]. The results of the scores of PCA of all three workflows are shown in Figure 2. The corresponding scree plots are shown in Figure S2. Regarding the variance of the first principal component (PC1), differences between the three workflows became visible. While PC1 accounts for 97% of variance in R, it dropped to 60.6% in CD when using a PhenylHexyl column in positive ionization mode. One explanation for this difference could be the different peak picking parameters. While the two open source workflows are highly adaptable methods regarding the optimization of parameters, CD is a black box with limited possibilities for optimization. Another explanation for the different PC1 could be that R and CD revealed a different amount of significant features. In contrast to the two open source workflows, CD did not detect any isotopes or adducts of the parent compound and its metabolites as significant features. This led to a low amount of compounds in relation to other substances within the incubation mixture and therefore, the variability between the group Blank and the groups Low and High are much higher. Figure S3 shows an example of the scores of PCA of the two open source workflows without isotopes and adducts using a PhenylHexyl column and ESI in positive ionization mode.

**Figure 3:**
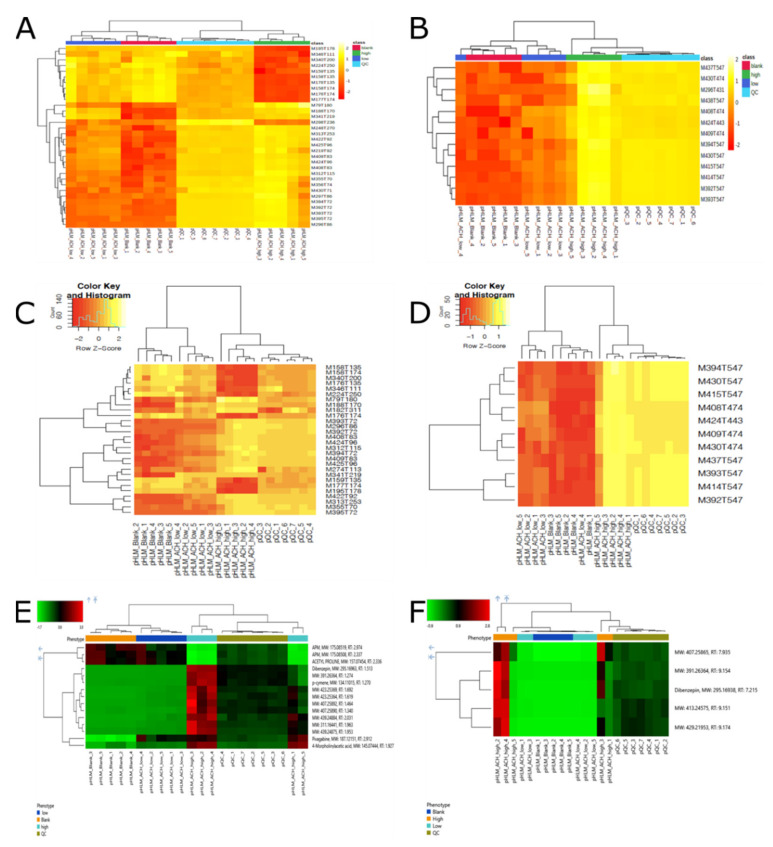
Heat map of hierarchical clustering. (**A** = XCMS Online/MetaboAnalyst, HILIC column, positive mode; **B** = XCMS Online/MetaboAnalyst, PhenylHexyl column, positive mode; **C** = R, HILIC column, positive mode; **D** = R, PhenylHexyl column, positive mode; **E** = Compound Discoverer, HILIC column, positive mode; **F** = Compound Discoverer, PhenylHexyl column, positive mode).

Another technique for statistical data analysis, which was used to assess the difference of the three workflows, was hierarchical clustering. Hierarchal cluster analysis refers to a specific family of distance-based procedures for cluster analysis. Clusters consist of objects that are less distant from each other than objects in other clusters. In untargeted metabolomic studies, heat maps of hierarchical clustering can be used to discover clustering patterns in the datasets. Figure 3 shows the resulting heatmaps for all three workflows. Except for the heatmap of CD when using a PhenylHexyl column, all other heatmaps showed a clear discrimination between samples from the group Blank and groups Low and High. Blank samples appear very close or within the cluster of samples from group Low. This could be explained by the concentration of the parent compound that was very low in group Low and therefore, this concentration could not sufficiently form as many metabolites as in group High. QC samples belonged to the cluster of samples from group High. Since pooled sample QC consisted of a mixture of every incubation sample, it contained the parent compound and its metabolites in a concentration of the samples from group High and Low. As shown in Figure 3F, only two samples of group High showed a clear discrimination between the other samples. The most likely explanation could be the low number of significant features. In comparison to the two open source workflows, CD detected only five significant features including the parent compound and four metabolites of A-CHMINACA, which showed their highest intensity in sample group High.

### 2.3. Targeted Metabolomics

#### 2.3.1. Identification of Significant Features

The results of the identification of significant features are summarized in Table 1 and Table 2. Annotated isotopes by CAMERA were not further analyzed. All other features were analyzed using the parallel reaction monitoring (PRM) method described below and the mass spectra are shown in Figure S4. Proposed structural formulas of the metabolites were deducted by comparing their spectra with those of the parent compound or reference spectra using the METLIN and Human Metabolome Database (HMBD) [25,26]. According to the Metabolomics Standards Initiative, this approach referred to category two, which means a putatively annotated compound [25]. It applies to all of the identified compounds except for 10 significant features, which are yet unknown and therefore, belongs to category four. Concerning the incubations using A-CHMINACA, significant features consisted of eight isotopes, two artifacts, nine metabolites, and four adducts. 

#### 2.3.2. Metabolism of A-CHMINACA in pHLM

**Figure 4:**
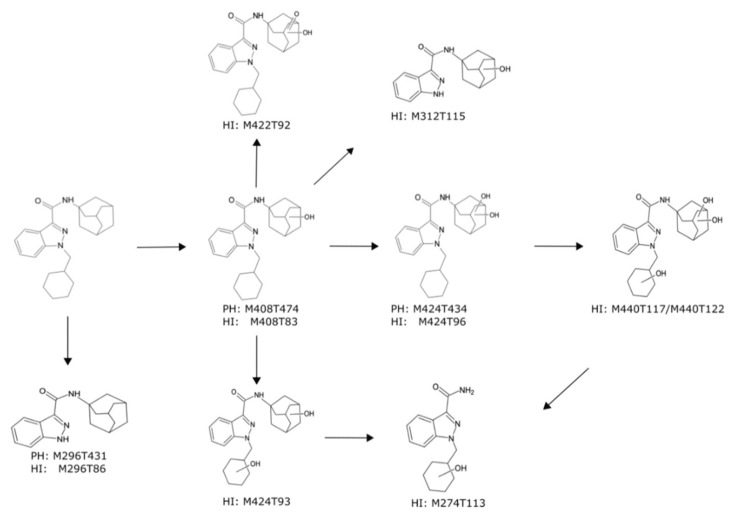
Metabolic pathways of A-CHMINACA in incubations with pooled human liver microsomes. Undefined hydroxylation position is indicated by unspecific bonds. Metabolites are annotated with their feature identity from untargeted metabolomics analysis. PH = PhenylHexyl column, HI = HILIC column.

From here onwards, only exact masses will be used for characterization of the parent compound and its respective metabolites. The proposed phase I metabolic pathways of A-CHMINACA in pHLM are summarized in Figure 4. After incubation with pHLM, nine metabolites were found in total. The main metabolic reaction was the hydroxylation of the adamantyl-ring, which has already been described for other synthetic cannabinoids containing such structure [27,28]. Protonated ions for hydroxylation were observed with *m*/*z* 408.2661 (C_25_H_34_N_3_O_2_), *m*/*z* 424.2610 (C_25_H_34_N_3_O_3_), and *m*/*z* 440.2565 (C_25_H_34_N_3_O_4_) corresponding to mono-, di-, and trihydroxylated derivates, respectively. Monohydroxylation of the adamantyl-ring concerning M408T83 (PH: M408T474) was identified by the occurrence of the highly abundant fragment ion with *m*/*z* 151.1117 (C_10_H_15_O) (Figure S4). Additionally, the occurrence of the fragment ion with *m*/*z* 133.1012 (C_10_H_13_), which resulted from water loss on the adamantyl-ring, supported this theory. M424T96 revealed an unmodified indazole-3-carbaldehyde moiety by the occurrence of the fragment ion with *m*/*z* 241.1335 (C_15_H_17_N_2_O), suggesting that this molecule was hydroxylated twice at the adamantyl-ring. The fragment ion with *m*/*z* 167.1067 (C_10_H_15_O_2_) also strongly indicated that both hydroxylations occurred at the adamantyl-ring. Since M440T117 and M440T122 had the same MS2 spectra, both gave rise to a fragment ion with *m*/*z* 422.2438 (C_25_H_32_N_3_O_3_) indicating the loss of water from the species with *m*/*z* 440.2544 (C_25_H_34_ N_3_O_4_). The observed fragment ion with *m*/*z* 167.1067 (C_10_H_15_O_2_) corresponded to the dihydroxylation at the adamantyl-ring. The loss of water at the adamantyl-ring resulted in a fragment ion with *m*/*z* 149.0961 (C_10_H_13_O). Whereas the monohydroxylation and dihydroxylation could be found significant by all three workflows, trihydroxylation was only found by CD. In comparison to M424T96 (PH: M424T434), the feature M424T93 gave rise to a fragment ion with *m*/*z* 151.1117 (C_10_H_15_O), indicating that hydroxylation occurred once at the adamantyl-ring. Hydroxylation and oxidation at the adamantyl-ring of M422T92 were identified by the occurrence of fragmentation with *m*/*z* 165.0910 (C_10_H_13_O_2_), while the fragment ion with *m*/*z* 404.2332 (C_25_H_30_ N_3_O_2_) resulted from water loss on the adamantyl-ring. Features M296T86 (PH: M296T431), M274T113, and M312T115 were formed after *N*-dealkylation of the indazole-3-carbaldeyde moiety, which was also described by Erratico et al. [29] in the in vitro metabolism of AB-CHMINACA. Using the current incubation conditions, no phase II metabolites were expected to be formed and were thus, not detected.

### 2.4. Comparison of the Three Software Workflows

Based on the usage of the three software workflows during this study and the results in the previous sections, an overview of the pros and cons concerning important criteria is given in Table 3. 

In comparison to the two open source workflows, the commercial software CD is characterized by its ease of use as a user-friendly black box. Thermo Fisher LC-HRMS/MS RAW files can be uploaded directly, and the desired workflow can be selected. The first results are available after a few mouse clicks. For statistical evaluation, only p-value and fold change have to be specified. Limitations for this kind of workflow are given by the preprocessing parameters, the normalization techniques, and the statistical analysis. Looking at the results in this study, this commercial software showed a low false positive rate for significant features, but neither isotopes nor adducts were detected that usually help in identifying significant features. Since CD is limited to its statistical test of Welch’s t-test, it does not allow one to do statistical evaluation of more than two groups and therefore, it is not a suitable workflow for complex datasets. The open source combination of XCMS Online and MetaboAnalyst 4.0 allowed for more intervention in the processing steps than CD. In XCMS Online, almost all parameters could be taken over with a few exceptions and MetaboAnalyst allowed a wide range of statistical tests. The report of MetaboAnalyst allowed an interpretation of the results. Both online tools were also based on the programming language R, but in contrast to the manually programmed R tool, less programming skills were required. The disadvantage is that due to the limited statistical test equipment of XCMS Online, a combination of these two online platforms was necessary. When comparing the results of the two open source workflows, both showed an almost identical false positive rate. The difference between the two workflows can only be seen in the user handling and the minimal difference in the number of significant features. The latter can be attributed to the not quite perfectly adjusted peak picking parameters using XCMS Online. 

In the case of complex datasets, the manually programmed R tool should be the best option. Due to its high number of packages, functions, and methods, it offers a great adaptability also with regard to statistical analysis. However, this open source workflow requires advanced programming skills. 

Regarding the results, the most relevant difference between the two open source workflows and the commercial software might be the optimization of the peak picking parameters. In contrast to the two open source workflows, the vendor-based software CD was used without changing any parameter (used as a black box as intended by the manufacturer). On the other hand, the two XCMS-based workflows are neutral for a broad range of data and therefore, they need parameter optimization because they are not usable under standard settings [15,19,20,21].

In summary, all three workflows have found the most important metabolites, but (toxico-)metabolomics includes not only exogenous metabolites but also endogenous ones. However, it must also be said that the investigated set was rather simple and less complex. Due to the minimal fluctuations, it was not necessary to normalize the dataset, for example, using an internal standard. This might be necessary when analyzing plasma or urine samples. The choice of the appropriate method should therefore depend on the complexity of the dataset and on previous knowledge. Complex datasets in this context mean that there are more than two groups in one study and that due to the complexity of the matrix, normalization to an endogenous biomarker is necessary. Previous knowledge basically means that the user has previously programmed with R or other programming languages. The manually programmed R tool required far more programming skills than the open source combination of XCMS Online and MetaboAnalyst 4.0. The commercial software CD on the other hand required almost no prior knowledge of metabolomics data processing. 

## 3. Materials and Methods 

### 3.1. Chemicals and Reagents

A-CHMINACA was provided by the EU project ADEBAR/State Bureau of Criminal Investigation Schleswig-Holstein (Kiel, Germany) for research purpose. The chemical purity and identity of the compound were verified by MS and nuclear magnetic resonance analysis. Ammonium formate, ammonium acetate, formic acid, isocitrate dehydrogenase, isocitrate, dipotassium phosphate, tripotassium phosphate, magnesium chloride, and superoxide dismutase were obtained from Sigma (Taufkirchen, Germany). Acetonitrile (LC-MS grade), methanol (LC-MS grade), and NADP-Na_2_ were from VWR (Darmstadt, Germany). pHLM (20 mg microsomal protein mL^−1^) was obtained from Corning (Amsterdam, The Netherlands). After delivery, pHLM were thawed at 37 °C, aliquoted, snap-frozen in liquid nitrogen, and stored at −80 °C until use.

### 3.2. pHLM Incubation

According to published procedures [16,30,31], incubations using pHLM were prepared as follows. A-CHMINACA was freshly dissolved in methanol and subsequently diluted with 100 mM phosphate buffer to obtain the required concentrations. Incubations were performed at 37 °C using final A-CHMINACA concentrations of 0 (Blank group), 5 (Low group), or 50 µM (High group) and 1 mg protein mL^−1^ pHLM. Final incubation mixtures also contained 90 mM phosphate buffer, 5 mM isocitrate, 5 mM Mg^2+^, 1.2 mM NADP^+^, 200 U mL^−1^ superoxide dismutase, and 0.5 U mL^−1^ isocitrate dehydrogenase. The final incubation volume was 50 µL. The reaction was stopped after 60 min by adding 50 µL of ice-cold acetonitrile and then, centrifugated for 2 min at 18,407× *g*. Every group consisted of five replicates. Pooled quality samples (QC group) were prepared by transferring 10 µL of each incubation into one MS vial. These were also used for optimization of the peak picking parameters, batch correction, and identification of significant features, as described below. An aliquot of 70 µL of the remaining supernatant was transferred into separate MS vials and used for metabolomics analysis, as described below.

### 3.3. LC-HRMS/MS Apparatus

In accordance with Manier et al. [16], analyses were performed by using a Thermo Fisher Scientific (TF, Dreieich, Germany) Dionex UltiMate 3000 RS pump consisting of a degasser, a quaternary pump, and an UltiMate Autosampler, coupled to a TF Q-Exactive Plus system including a heated electrospray ionization (HESI)-II source. Prior to every experiment, the performance of the columns and mass spectrometer was tested using a test mixture as described by Maurer et al. [32,33]. Gradient normal phase elution was performed on a Macherey-Nagel (Düren, Germany) HILIC Nucleodur column (125 mm × 3 mm, 3 µm) and reversed phase elution using a TF Accucore PhenylHexyl column (100 mm × 2.1 mm, 2.6 µm). The mobile phase and gradient for the PhenylHexyl column consisted of 2 mM aqueous ammonium formate containing acetonitrile (1%, *v*/*v*) and formic acid (0.1%, *v*/*v*, pH 3, eluent A), as well as 2 mM ammonium formate solution with acetonitrile:methanol (1:1, *v*/*v*) containing water (1%, *v*/*v*) and formic acid (0.1%, *v*/*v*, eluent B). The flow rate was set from 1–10 min to 500 µL min^−1^ and from 10–13.5 min to 800 µL min^−1^ using the following gradient: 0–1.0 min hold 99% A, 1–10 min to 1% A, 10–11.5 min hold 1% A, 11.5–13.5 min hold 99% A. The gradient elution for normal phase chromatography was performed using aqueous ammonium acetate (200 mM, eluent C) and acetonitrile containing formic acid (0.1%, *v*/*v*, eluent D). The flow rate was set to 500 µL × min^−1^ using the following gradient: 0–1 min hold 2% C, 1–5 min to 20% C, 5–8.5 min to 60% C, 8.5–10 min hold 60% C, 10–12 min hold 2% C. For preparation and cleaning of the injection system, isopropanol:water (90:10, *v*/*v*) was used. Due to the lipophilic properties of A-CHMINACA, eluent D was used for the flushing of both columns. The following settings were used: wash volume, 100 µL; wash speed, 4000 nL s^−1^; loop wash factor, 2. Column temperature for every analysis was set to 40 °C, maintained by a Dionex UltiMate 3000 RS analytical column heater. Injection volume was set to 1 µL. HESI-II source conditions were as follows: ionization mode, positive or negative; sheath gas, 60 AU; auxiliary gas, 10 AU; sweep gas, 3 AU; spray voltage, 3.5 kV in positive and −4.0 kV in negative mode; heater temperature, 320 °C; ion transfer capillary temperature, 320 °C; and S-lens RF level, 50.0. Mass spectrometry for untargeted metabolomics was performed according to a previously optimized workflow [15,16]. The settings for full scan (FS) data acquisition were as follows: resolution, 140,000 fwhm; microscan, 1; automatic gain control (AGC) target, 5 × 10^5^; maximum injection time, 200 ms; scan range, *m*/*z* 50–750; spectrum data type; centroid. Significant features were subsequently identified using PRM. Settings for PRM data acquisition were as follows: resolution, 70,000 fwhm; microscans, 1; AGC target, 5 × 10^5^; maximum injection time, 200 ms; isolation window, 0.4 *m*/*z*; collisions energy (CE), 10, 20, 30, or 40 eV; spectrum data type, centroid. The inclusion list contained the monoisotopic masses of all significant features and a time window of their retention time ±60 s. TF Xcalibur software version 3.0.63 was used for data handling. Due to the carry-over effect of A-CHMINACA, the analysis was performed using the following sequence order: five injections of eluent D samples at the beginning of the sequence for apparatus equilibration, followed by five injections of pooled QC samples, five blank groups, five low groups, and five high groups. Additionally, one QC injection was performed every five samples to monitor batch effects, as described by Wehrens et al. [34].

### 3.4. Dataset Processing with Different Software

For the two open source software workflows, Proteo Wizard was used to convert Thermo Fisher LC-HRMS/MS RAW files into mzXML files [35]. Optimization of the XCMS parameters was done by using a comprehensive parameter sweeping approach [15]. Table S1 summarizes the peak picking and alignment parameters used for the two open source workflows.

In the case of using R, peak picking was performed using XCMS in an R environment [14,36] and the R package CAMERA [37] was used for the annotation of isotopes, adducts, and artifacts. The dataset was filtered keeping merely those features with a p-value using Bonferroni correction [23]. Feature abundances with a value of zero were replaced by the lowest measured abundance as a surrogate limit of detection and the whole dataset was subsequently log10 transformed [34]. Batch correction was performed for those features that were detected in every QC sample. Corresponding feature abundances were corrected using a linear model to extrapolate abundance drift between QC samples [34]. Principal component analysis (PCA) and hierarchical clustering were used to investigate patterns in the dataset. Names for the features were adopted from XCMS using “M” followed by rounded mass and “T” followed by the retention time in seconds. The R script and the mzXML files can be found at https://github.com/sehem/HLM_Metabolomics.git. 

For the combination of XCMS Online and MetaboAnalyst 4.0, first, XCMS Online was used for peak picking and alignment using the optimized parameters listed in Table S1. The resulted table of XCMS Online was then processed by removing all features under a retention time of 1 min and above 10 min and all columns were removed except the peak areas of each feature in each sample. The modified table was then uploaded to MetaboAnalyst 4.0 for statistical analysis. For normalization of the dataset, the following settings were used: sample normalization, none; data scaling, none; and data transformation, log transformation. Subsequently, one-way ANOVA was selected using Bonferroni correction for *p*-value. To investigate patterns in the dataset, PCA and hierarchical clustering using heat maps and dendrograms were selected. For hierarchical clustering, distance measures using Euclidean distances and clustering algorithms using complete were chosen.

In the case of CD, Thermo Fisher LC-HRMS/MS RAW files were uploaded and definitions of study factors in the form of categorical factors were entered. Subsequently, the ratios blank/low, blank/high, and low/high were defined. Afterwards, a predefined untargeted workflow named “Untargeted Metabolomics with statistics detect unknowns with ID using Online Database and mzLogic” was used. This workflow included findings and identified the differences between samples, performed retention time alignment, identified compounds using mzCloud, ChemSpider, and calculated differential analysis such as ANOVA, determined *p*-values, and fold changes. Bonferroni correction for *p*-value and fold-change of 1 were used for ANOVA.

### 3.5. Identification of Significant Features

Identification of significant features was done by recording MS/MS spectra using the PRM method mentioned above. Spectra were imported to NIST MSSEARCH version 2.3, after conversion to mzXML format using ProteoWizard [35]. According to Manier et al. [17], a library search for identification was conducted using the following settings: spectrum search type, identity (MS/MS); precursor ion *m*/*z*, in spectrum; spectrum search options, none; presearch, off; other options, none. MS/MS search was conducted using the following settings: precursor tolerance, ±5 ppm; product ion tolerance, ±10 ppm; ignoring peaks around precursor, ±*m*/*z* 1. The search was conducted by using the following libraries: NIST 14 (nist_msms and nist_msms2 sublibraries) and Wiley METLIN Mass Spectral Database. Metabolites of the investigated synthetic cannabinoid A-CHMINACA were tentatively identified by interpreting their spectra in comparison to that of the parent compound.

## 4. Conclusions 

In this study, a dataset of pHLM incubations of the synthetic cannabinoid A-CHMINACA was used to evaluate data processing of three different software workflows under their respective optimal parameter settings. The commercial software CD is a vendor-based software, which was specifically developed for the type of MS instrument used in this study. The two open source workflows, XCMS Online/MetaboAnalyst and R, both use the “gold standard” XCMS for peak picking and alignment for untargeted metabolomics data evaluation after LC-HRMS/MS analysis.

While the two open source workflows were highly adaptable methods regarding the optimization of parameters, CD is a user-friendly black box with limited possibilities for optimization. Additionally, the metabolic profile of A-CHMINACA in pHLM was determined to compare the three software solutions. The main metabolic reactions were the hydroxylation of the adamantyl-ring and *N*-dealkylation of the indazole-3-carbaldeyde moiety.

In relation to the results of this study, CD as an all-in-one solution is characterized by its ease of use and therefore, seems suitable for simple and small metabolomic studies, as the dataset used in this study. However, it is not possible to use the right statistical test, since the dataset exists of three groups. Taking this into account, the statistical results of the used dataset can be better represented with the two open source workflows. Both open source workflows allowed extensive customization but particularly in the case of R, advanced programming skills are required, while XCMS Online/MetaboAnalyst is an almost entirely point-and-click experience. Nevertheless, both provided high flexibility and may be suitable for more complex studies and questions. The metabolic fate of A-CHMINACA in pHLM was identified best by the two open source workflows.

## Figures and Tables

**Table 1:** Overview of significant features of A-CHMINACA after untargeted analysis using reversed-phase chromatography in positive mode of all three workflows.

Feature	Measured Mass, *m*/*z*	Retention Time, s	Found with	Identity
M296T431	296.1768	431	XM, CD	**A-CHMINACA-M (*N*-dealkyl-)**
M424T443	424.2610	443	R, XM	**A-CHMINACA-M (di-HO-)**
M408T474	408.2661	474	R, XM, CD	**A-CHMINACA-M (HO-)**
M409T474	409.2693	474	R, XM	A-CHMINACA-M (HO-) ^13^C isotope
M430T474	430.2481	474	R, XM	A-CHMINACA-M (HO-) adduct [M + Na]^+^
M392T547	392.2710	547	R, XM, CD	A-CHMINACA
M393T547	393.2743	547	R, XM	A-CHMINACA ^13^C isotope
M394T547	394.2775	547	R, XM	A-CHMINACA ^13^C_2_ isotope
M414T547	414.2530	547	R, XM, CD	A-CHMINACA adduct [M + Na]^+^
M415T547	415.2562	547	R, XM	A-CHMINACA adduct [M + Na]^+ 13^C isotope
M430T547	430.2270	547	R, XM, CD	A-CHMINACA adduct [M + K]^+^
M437T547	437.3290	547	R, XM	A-CHMINACA adduct
M438T547	438.3320	547	XM	A-CHMINACA adduct ^13^C isotope

Features are ordered by retention time and *m*/*z*. Isotopes were annotated by the R package CAMERA and not further identified. Metabolites are indicated by bold font. XM = XCMS Online/MetaboAnalyst, CD = Compound Discoverer.

**Table 2:** Overview of significant features of A-CHMINACA after untargeted analysis using a normal phase (HILIC) column in positive mode of all three workflows.

Feature	Measured Mass, *m*/*z*	Retention Time, s	Found with	Identity
M355T70	355.2392	70	R, XM	Unknown
M430T71	430.2270	71	XM	A-CHMINACA adduct [M + K]^+^
M392T72	392.2710	72	R, XM, CD	A-CHMINACA
M393T72	393.2743	72	R, XM	A-CHMINACA ^13^C isotope
M394T72	394.2775	72	R, XM	A-CHMINACA ^13^C_2_ isotope
M395T72	395.2809	72	R, XM	A-CHMINACA ^13^C_3_ isotope
M356T74	356.1802	74	XM	Unknown
M135T76	135.1174	76	CD	A-CHMINACA artifact (adamantyl-ring)
M408T83	408.2661	83	R, XM, CD	**A-CHMINACA-M (HO-)**
M409T83	409.2693	83	R, XM	A-CHMINACA-M (HO-) ^13^C isotope
M296T86	296.1768	86	R, XM, CD	**A-CHMINACA-M (** ***N*** **-dealkyl-)**
M297T86	297.1800	86	XM	A-CHMINACA-M (*N*-dealkyl-) ^13^C isotope
M408T88	408.2661	88	CD	**A-CHIMINACA-M (HO-)**
M422T92	422.2453	92	R, XM	**A-CHIMINACA-M (HO, Oxo)**
M424T93	424.2610	93	CD	**A-CHMINACA-M (di-HO-)**
M424T96	424.2610	96	R, XM, CD	**A-CHMINACA-M (di-HO-)**
M425T96	425.2644	96	R, XM	A-CHMINACA-M (di-HO-) ^13^C isotope
M274T113	274.1559	113	R, XM	**A-CHMINACA-M (HO-) (*N*-dealkyl-)**
M312T115	312.1715	115	R, XM, CD	**A-CHMINACA-M (HO-) (** ***N*** **-dealkyl-)**
M146T116	146.0819	116	CD	A-CHMINACA artifact (indazole-core)
M440T117	440.2561	117	CD	**A-CHMINACA-M (tri-HO-)**
M440T122	440.2565	122	CD	**A-CHMINACA-M (tri-HO-)**
M176T135	176.0924	135	R, XM, CD	Unknown
M158T135	158.0818	135	R, XM, CD	[M + H − H_2_O]+175.086
M188T170	188.1288	170	R, XM, CD	Unknown
M158T174	158.0818	174	R, XM	[M + H − H_2_O]+175.086
M176T174	176.0924	174	R, XM, CD	Unknown
M341T219	341.2447	219	R, XM	Unknown
M313T253	313.2649	253	R, XM	Unknown
M248T270	248.2382	270	XM	Unknown

Features are ordered by retention time and *m*/*z*. Isotopes were annotated by the R package CAMERA and not further identified. Metabolites are indicated by bold font. XM = XCMS Online/MetaboAnalyst, CD = Compound Discoverer.

**Table 3:** Overview of important criteria by which the three workflows can be classified.

Criteria	Compound Discoverer	XCMS Online/MetaboAnalyst 4.0	Manually Programmed R Tool
Open source	-	+	+
Low false-positive rate	+	-	+
Flexibility	-	-/+	+
Complex datasets	-	+	+
Using raw data	+	-	-
Required prior knowledge	-	-	+
Annotation of isotopes and adducts	-	+	+

Evaluation criteria: + = available/good; - = not available/bad.

## Supplementary Materials

The supplementary materials are available online at https://www.mdpi.com/2218-1989/10/9/378/s1.
