# Addendum: Hemmer, S., et al. Comparison of Three Untargeted Data Processing Workflows for Evaluating LC-HRMS Metabolomics Data. *Metabolites* 2020, *10*, 378

**DOI:** 10.3390/metabo10110432

**Published:** 2020-10-27

**Authors:** Selina Hemmer, Sascha K. Manier, Svenja Fischmann, Folker Westphal, Lea Wagmann, Markus R. Meyer

**Affiliations:** 1 Department of Experimental and Clinical Toxicology, Institute of Experimental and Clinical Pharmacology and Toxicology, Center for Molecular Signaling (PZMS), Saarland University, 66421 Homburg, Germany; selina.hemmer@uks.eu (S.H.); Sascha.manier@uks.eu (S.K.M.); lea.wagmann@uks.eu (L.W.); 2 State Bureau of Criminal Investigation Schleswig-Holstein, 24116 Kiel, Germany; Svenja.Dr.Fischmann@polizei.landsh.de (S.F.); Folker.Dr.Westphal@polizei.landsh.de (F.W.)

The authors wish to make the following comment to this paper [1]. 

To avoid any misunderstandings and misleading interpretations regarding the general possibilities of Compound Discoverer (CD) when reading the paper, we would like to add the following comment. We used Compound Discoverer (CD) with an already existing workflow for untargeted metabolomics namely “Untargeted Metabolomics with statistics detect unknowns with ID using Online Database and mzLogic” without changing any parameters (“out-of-the-box”). Therefore, some features of CD were not used, such as direct evaluation of isotopes and adducts, Scripting node for normalization, and comparing three groups visually after ANOVA, as it was carried out for the other two workflows.

Readers should be aware that CD is nevertheless able to determine isotopic patterns and elemental composition, integrate Scripting node that can then be used to integrate R or Python scripts, and is capable of comparing multiple groups, performing ANOVA with Tukey as a post-hoc test, and nested designs. 

The authors would like to apologize for any misunderstandings appearing from the original manuscript. These comments do not affect the scientific results. 
