# Supplementary material for: Comparison of Three Untargeted Data Processing Workflows for Evaluating LC-HRMS Metabolomics Data
Source: Metabolites. 2020 Sep 21;10(9):378. doi: 10.3390/metabo10090378 (PMC7570355; doi:10.3390/metabo10090378)
Supplement: Supplementary file 1 [file metabolites-10-00378-s001.pdf]

Supplementary materials

Comparison of Three Untargeted Data Processing Workflows for Evaluating LC-HRMS Metabolomics Data

Selina Hemmer [1], Sascha K. Manier [1], Svenja Fischmann [2], Folker Westphal [2], Lea Wagmann [1] and Markus R. Meyer [1,*]

[1] Department of Experimental and Clinical Toxicology, Institute of Experimental and Clinical Pharmacology and Toxicology, Center for Molecular Signaling (PZMS), Saarland University, 66421 Homburg, Germany; selina.hemmer@uks.eu (S.H.); sascha.manier@uks.eu (S.K.M.); lea.wagmann@uks.eu (L.W.)

[2] State Bureau of Criminal Investigation Schleswig-Holstein, 24116 Kiel, Germany; Svenja.Dr.Fischmann@polizei.landsh.de (S.F.); Folker.Dr.Westphal@polizei.landsh.de (F.W.)

* Correspondence: markus.meyer@uks.eu

Table S1. Peak picking and alignment parameters used for preprocessing using R and XCMS Online.

Column	Polarity	Peakwidth, min	Peakwidth, max	ppm	Snthresh	Mzdiff	Prefilter 1	Prefilter 2	bw
**HI**	pos	10	22	1.0	39	0.07	7	900	0.1 (1*)
**HI**	neg	10	12	1.9	69	0.016	5	6100	0.1 (1*)
**PH**	pos	9.3	12	1.2	83	0.02	10	5400	0.1 (1*)
**PH**	neg	9.3	12	1.0	96	0.068	6	8200	0.5 (1*)

HI = HILIC, PH = PhenylHexyl, pos = positive, neg = negative, ppm = allowed ppm deviation of mass traces for peak picking, snthresh = signal to noise threshold, mzdiff = minimum difference in m/z for two peaks to be considered as separate, prefilter 1 = minimum of scan points, prefilter 2 = minimum abundance, bw = bandwidth for grouping of peaks across separate chromatograms, * value used for XCMS Online.

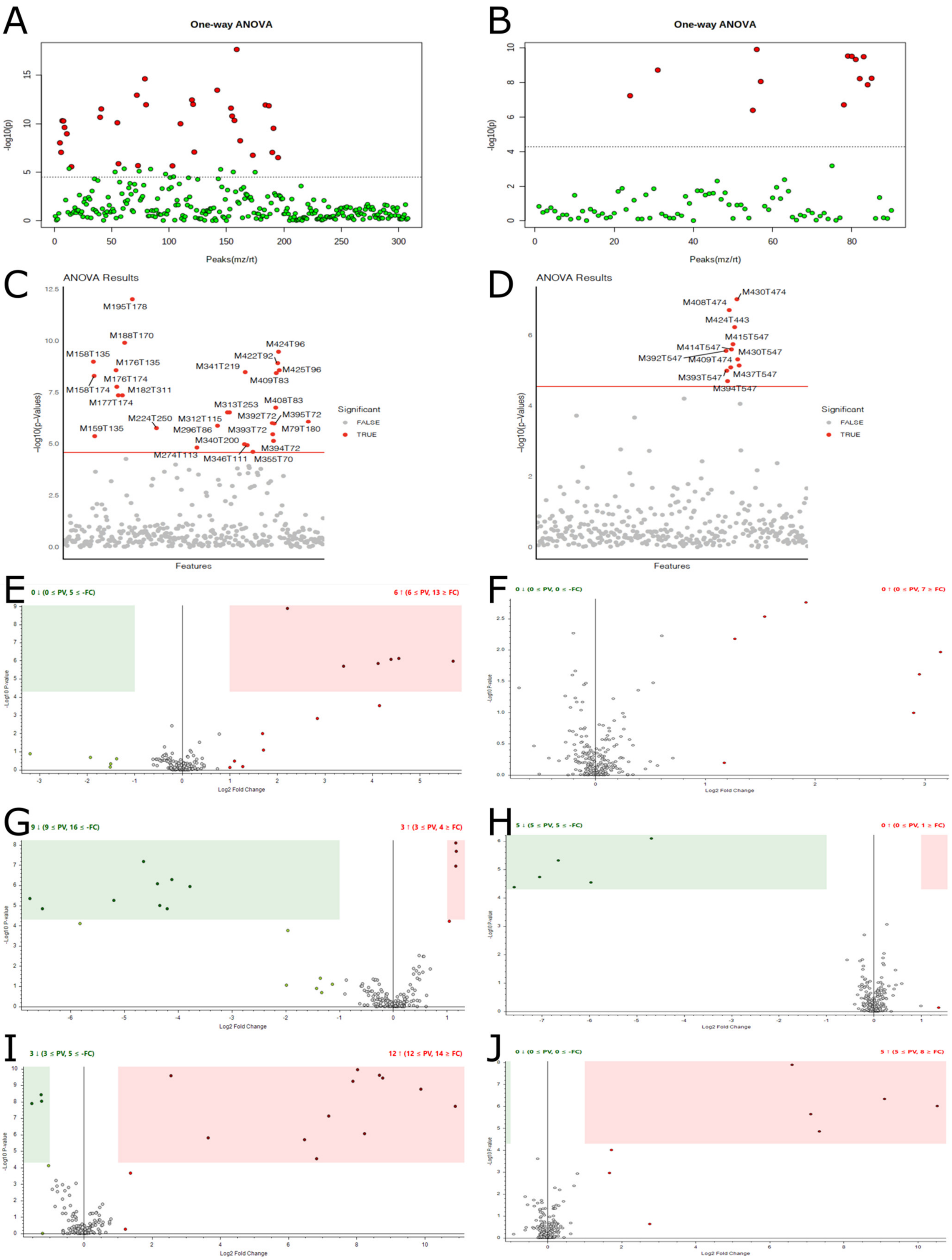

Figure S1. Results of one-way ANOVA for A-CHMINACA incubations analyzed in positive ionization mode. **A** = XCMS Online/MetaboAnalyst, HILIC column; **B** = XCMS Online/MetaboAnalyst, PhenylHexyl column; **C** = R, HILIC column; **D** = R, PhenylHexyl column; **E** = Compound Discoverer, Low and Blank, HILIC column; **F** = Compound Discoverer, Low and Blank,

PhenylHexyl column; **G** = Compound Discoverer, Low and High, HILIC column; **H** = Compound Discoverer, Low and High, PhenylHexyl column; **I** = Compound Discoverer, High and Blank, HILIC column; **J** = Compound Discoverer, High and Blank, PhenylHexyl column.

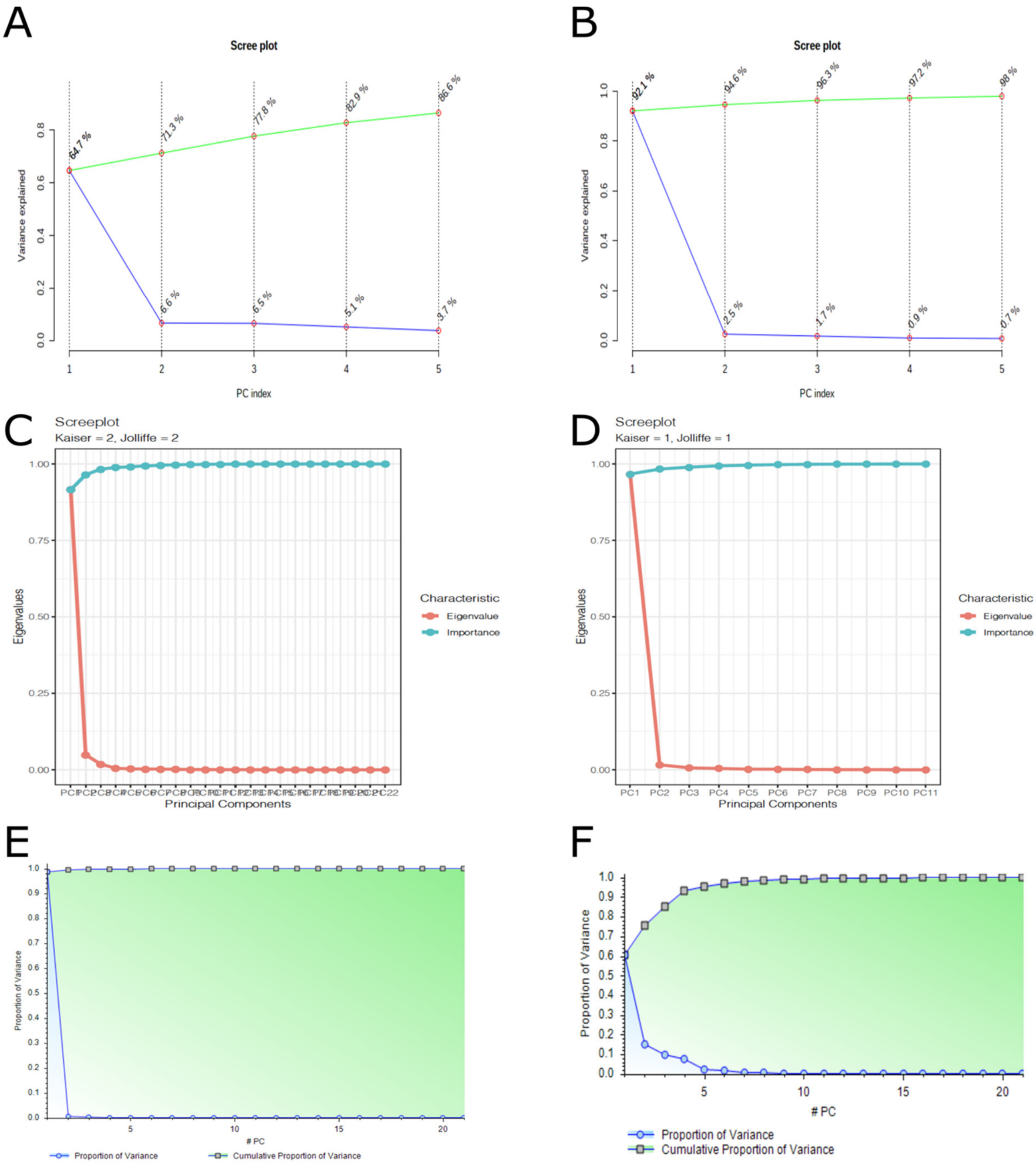

Figure S2. Results of scree plots for A-CHMINACA incubations analyzed in positive ionization mode. **A** = XCMS Online/MetaboAnalyst, HILIC column; **B** = XCMS Online/MetaboAnalyst, PhenylHexyl column; **C** = R, HILIC column; **D** = R, PhenylHexyl column; **E** = Compound Discoverer, HILIC column; **F** = Compound Discoverer, PhenylHexyl column.

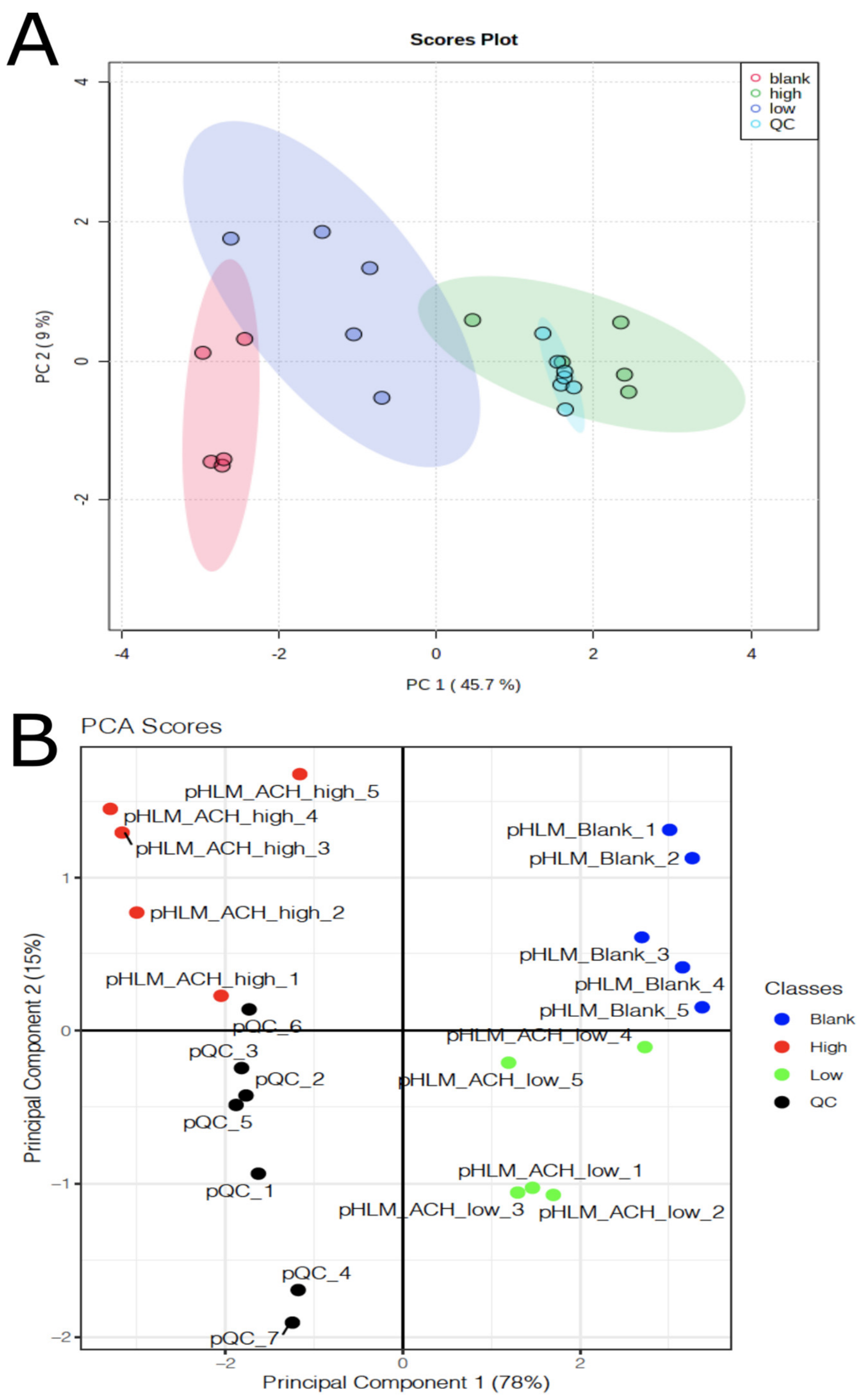

Figure S3. Results of scores of principal component analysis for A-CHMINACA incubations analyzed in positive ionization mode without isotopes and adducts. **A** = XCMS Online/MetaboAnalyst, PhenylHexyl column; **B** = R, PhenylHexyl column.

M392T72, A-CHMINACA

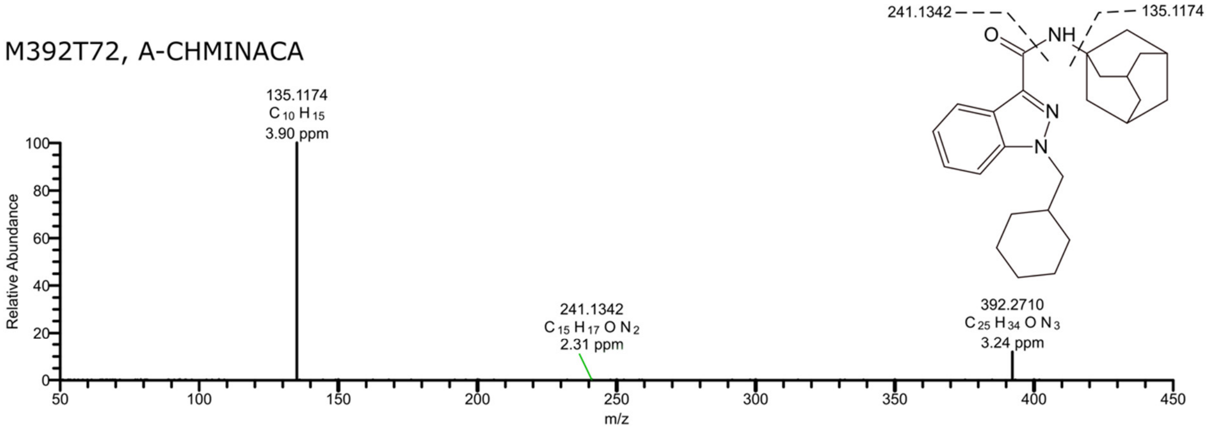

M296T86, A-CHMINACA (*N*-dealkyl-)

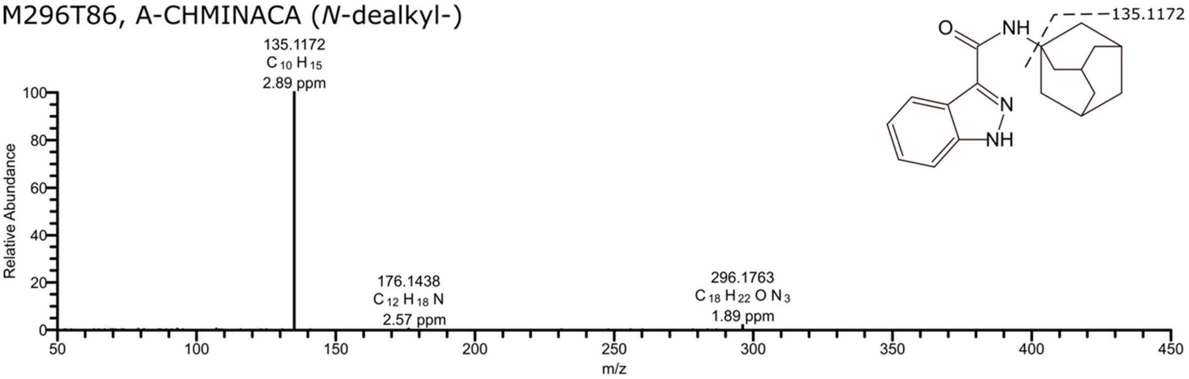

M408T83, A-CHMINACA-M (HO-)

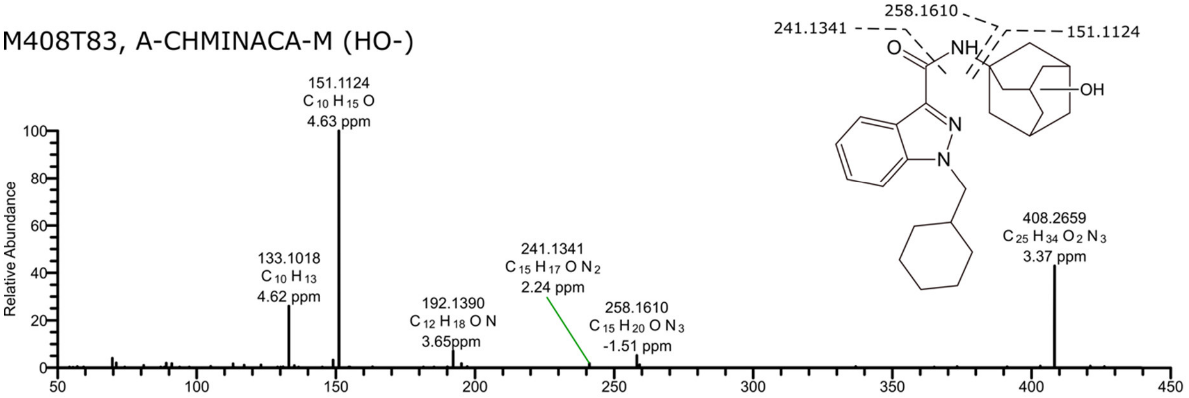

M422T92, A-CHMINACA-M (HO, Oxo)

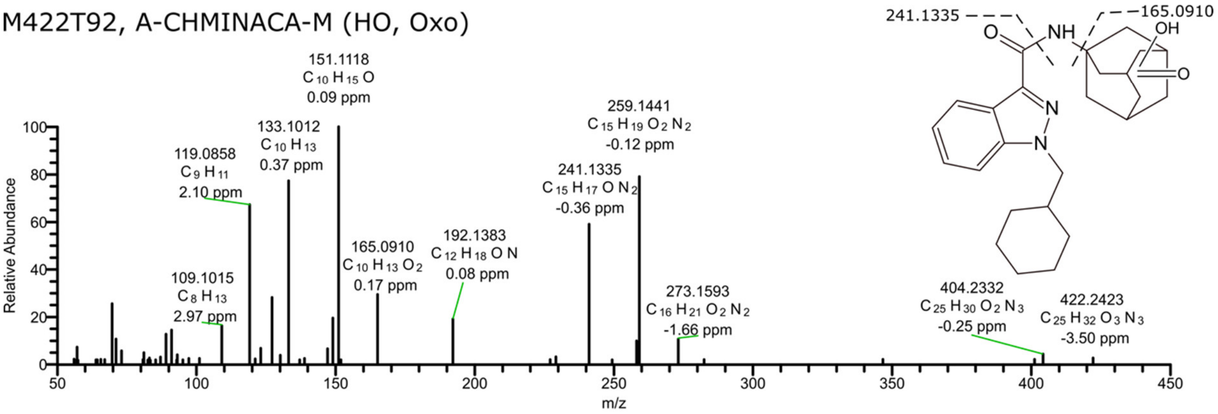

M424T96, A-CHMINACA-M (di-HO-)

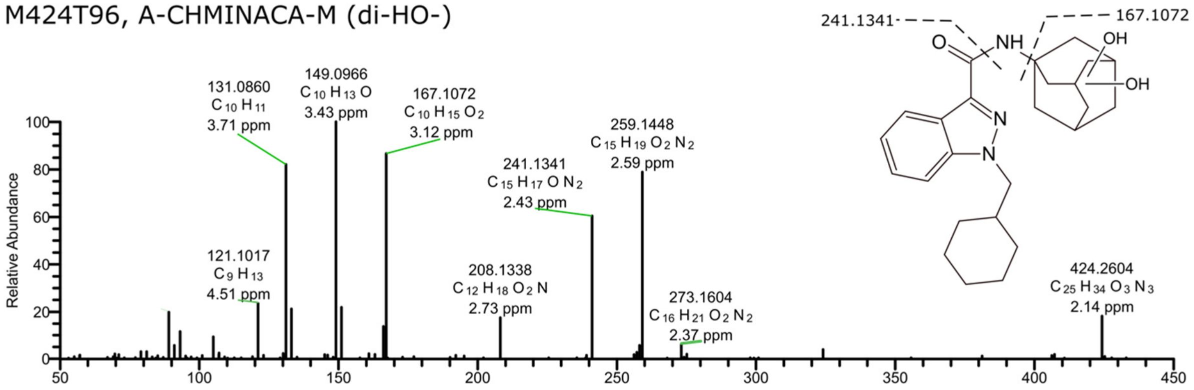

M424T93, A-CHMINACA-M (di-HO-)

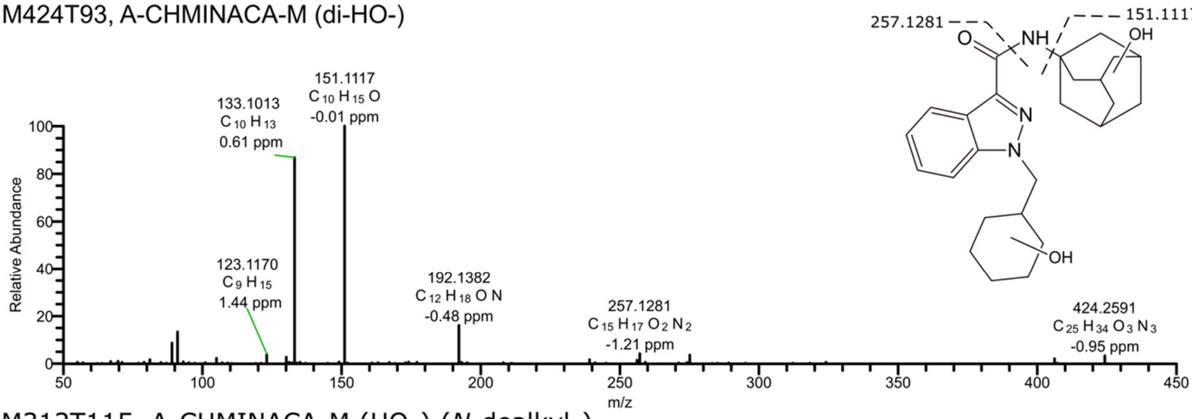

M312T115, A-CHMINACA-M (HO-) (*N*-dealkyl-)

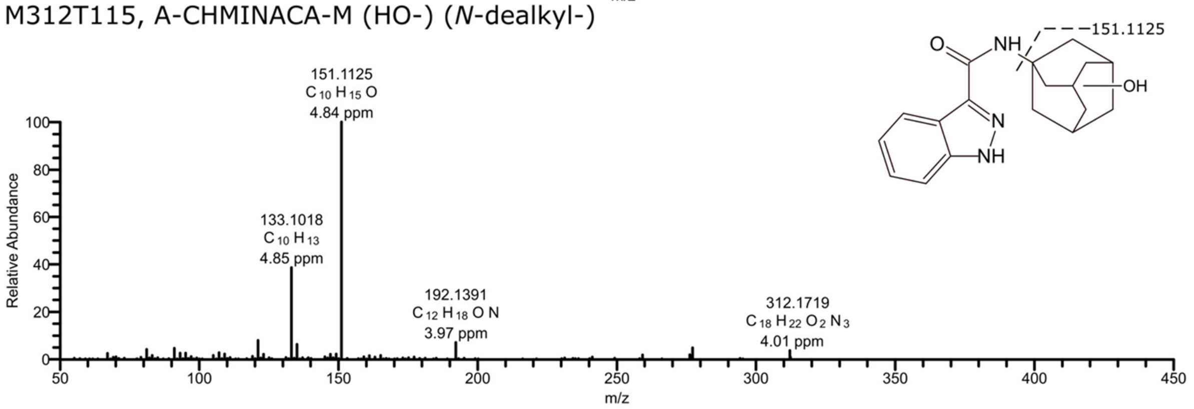

M274T113, A-CHMINACA-M (HO-) (*N*-dealkyl-)

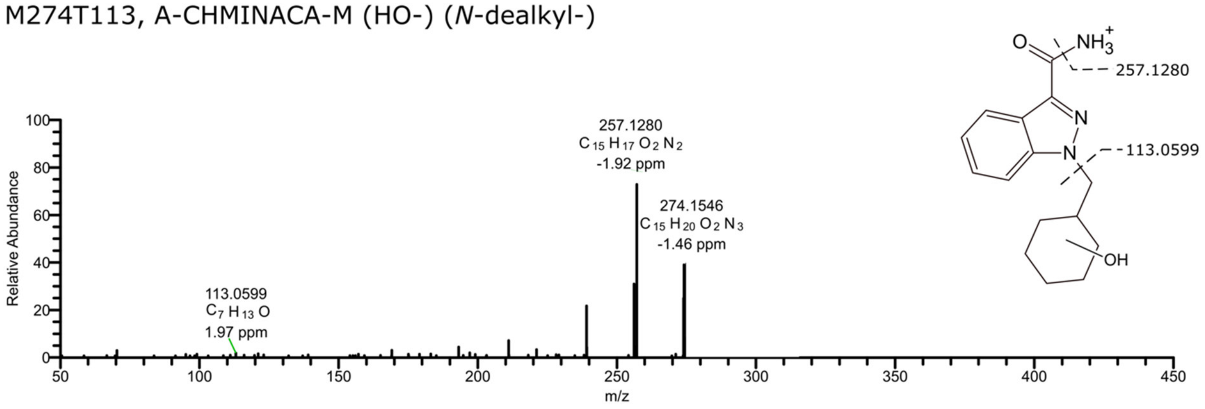

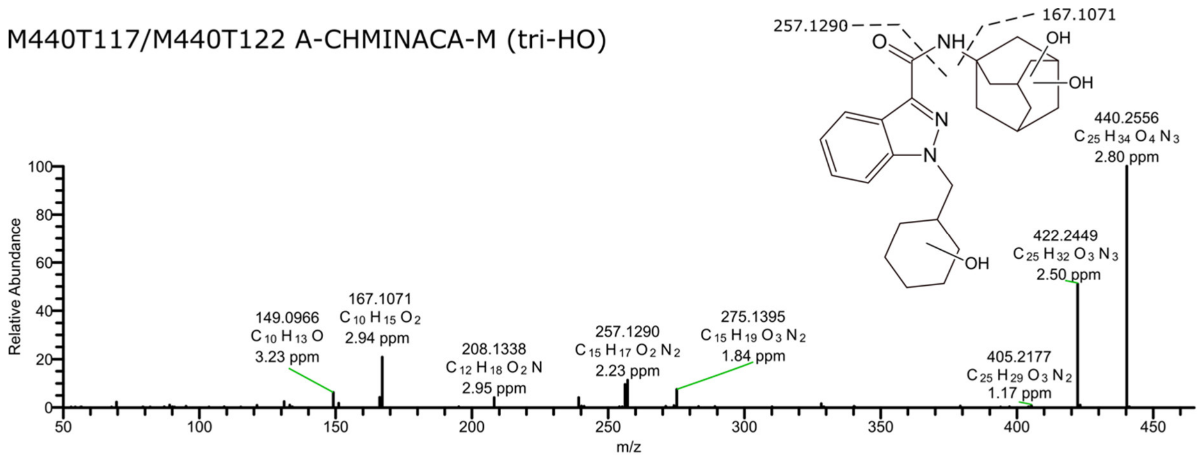

Figure S4. LC-HRMS/MS spectra of significant features in A-CHMINACA incubations analyzed with a HILIC column in positive ionization mode. Fragments with accurate mass, calculated elemental formula, and mass error value in parts per million (ppm).
